# DEF6 expression in ovarian carcinoma correlates with poor patient survival

**DOI:** 10.1186/s13000-016-0518-y

**Published:** 2016-08-03

**Authors:** Phui-Ly Liew, Chih-Yeu Fang, Yu-Chieh Lee, Yi-Chih Lee, Chi-Long Chen, Jan-Show Chu

**Affiliations:** 1 Department of Pathology, Shuang Ho Hospital, Taipei Medical University, New Taipei City, 23561 Taiwan; 2 Department of Pathology, School of Medicine, College of Medicine, Taipei Medical University, No. 250, Wu Xing Street, Taipei, 11031 Taiwan; 3 Department of Pathology, Wan Fang Hospital, Taipei Medical University, Taipei, 116 Taiwan; 4 Graduate Institute of Medical Sciences, College of Medicine, Taipei Medical University, Taipei, 11031 Taiwan; 5 Department of International Business, Chien Hsin University of Science and Technology, Taoyuan, 32097 Taiwan; 6 Department of Pathology, Taipei Medical University Hospital, Taipei Medical University, Taipei, 11031 Taiwan

**Keywords:** Ovarian carcinoma, DEF6, p16, Prognosis

## Abstract

**Background:**

Increased expression of DEF6 is correlated with the malignant behavior of various cancers. Both DEF6 and p16 contribute to the regulation of cell cycle progression, and p53 plays important role in the cell cycle checkpoints. This study was designed to elucidate the prognostic significance of DEF6, p53 and p16 immunoexpressions in different histology subtypes of ovarian carcinoma.

**Methods:**

Immunohistochemistry results of DEF6, p53 and p16 on ovarian carcinoma were compared with histology subtypes, clinical data, overall survival (OS) and disease-free survival (DFS) by Cox regression and Kaplan-Meier analysis.

**Results:**

We studied 180 cases of ovarian carcinomas (75 high-grade serous, 41 clear cell, 36 mucinous and 28 endometrioid), including 109 FIGO stage I-II cases and 71 FIGO stage III-IV cases. Ovarian carcinomas positive for both DEF6 and p16 expression were associated with the worst OS (*P* = 0.027) and DFS (*P* = 0.023), whereas those negative for both DEF6 and p16 had the best OS and DFS. Aberrant p53 expression combined with positive DEF6 was associated with worst OS (*P* = 0.031) and DFS (*P* = 0.028). Kaplan-Meier analysis showed that significantly shorter survival rates were seen in patients with high expressions of DEF6 (*P* = 0.008) and p16 (*P* = 0.022). Patients with aberrant p53 expression in high-grade serous carcinoma (*P* = 0.012) and patients with high DEF6 expression in clear cell carcinoma (*P* = 0.001) were also associated with shorter overall survival. In univariate analysis, FIGO stage, DEF6 and p16 were associated with poor prognosis. DEF6 expression was the only independent prognostic factor correlated with shorted OS (HR 2.115; *P* = 0.025) and DFS (HR 2.248; *P* = 0.016) upon multivariate analysis.

**Conclusions:**

DEF6 expression may serve as an independent prognostic factor, and interacted positively with p16 toward high tumor stage and shorter survival.

**Electronic supplementary material:**

The online version of this article (doi:10.1186/s13000-016-0518-y) contains supplementary material, which is available to authorized users.

## Background

Ovarian carcinoma is the most lethal malignancy of the female genital tract, mainly due to the failure of early diagnosis, heterogeneous histology subtypes and the limitations for the conventional chemotherapies [1, 2]. The important prognostic factors include tumor stage, age at initial diagnosis, tumor morphological subtypes and grade, optimal resection for advanced ovarian cancer, as well as the effect of chemotherapy following primary surgery [3]. Current researches have focused on the study of various molecular signaling reactions or pathways in ovarian carcinoma to explore the molecular markers for early detection, prognosis assessment and hopefully as potential therapeutic targets.

Interferon regulatory factor 4 binding protein (IBP, also known as: DEF6) first identified in 2003 [4], plays multiple important roles in various biological processes that involve the immune system. Loss of DEF6 in mice resulted in the development of systemic autoimmunity and developmental defects at the earliest stage of thymocyte differentiation [5–10]. Besides, DEF6 plays important role in the regulation of cell motility, cytoskeletal rearrangements, focal complex/adhesion assembly, cell polarity and cell migration through the stimulation of actin polymerization [11–17].

Signaling involving DEF6 has been implicated in tumorigenesis. Increased expression of DEF6 has been shown to be correlated with the malignant behavior of extra-skeletal myxoid chondrosarcoma [18], colorectal cancer [19], breast cancer cells [20], and oral squamous cell carcinoma [21]. DEF6 may serve as a potential target for anti-angiogenic intervention in renal cell carcinoma [22]. DEF6 is also a novel target of tumor suppressor p53 and can suppress cisplatin-mediated apoptosis of breast cancer cells via a negative feedback regulation of the p53 signaling pathway [23]. High levels of DEF6 were found to decrease cisplatin-induced growth suppression and apoptotic cell death, in association with decreased p53 activity and imbalanced expressions of the Bcl-2 family members. Recently, p16 has been shown as a prognostic indicator in ovarian/fallopian tubal high-grade serous carcinoma [24–26]. Moreover, both DEF6 and p16 contribute to the regulation of cell cycle progression, and p53 plays important role in the cell cycle checkpoints. Despite their potential close interaction in cell cycle progression, the roles of DEF6, p16 and p53 have not been fully elucidated in ovarian carcinomas.

In the present study, we sought to better understand the differential expression of DEF6, its relation to the expressions of p53 and p16, and the prognostic significance in different histology subtypes of ovarian carcinoma based on a well-defined cohort of ovarian carcinomas. Clinicopathological data and survival curves were compared between patients with different scores of DEF6 to explore the potential of DEF6 as a prognostic marker.

## Methods

### Patient specimens

We collected cases with surgically operated ovarian carcinomas from the files of Departments of Pathology of Taipei Medical University Hospital and Wan Fang Medical Center between January 1998 and December 2011. We used the diagnostic criteria of the World Health Organization classification [1] and tumor staging system of the International Federation of Gynecology and Obstetrics (FIGO). The pathological diagnosis was reviewed by at least two pathologists. Cases of secondary metastasis to the ovaries were excluded.

Pathologic variables included histology subtypes (i.e. low- or high-grade serous carcinoma, mucinous carcinoma, endometrioid carcinoma and clear cell carcinoma), stage, therapy, recurrence free interval (if applicable), and site of recurrent disease (if applicable). Debulking surgery was found to be optimal if the maximum diameters of the individual residual tumor deposits were all less than 1.0 cm. Only cases with optimal resection were enrolled and analyzed in this study. Patient information were de-identified and assigned a study number. Tissue microarrays (TMA) with three to four tumor regions were chosen from paraffin embedded blocks. Each tumor core measured 0.3 cm in maximal diameter. This study had been reviewed and approved by the Institutional Review Board of Taipei Medical University (TMU-IRB 99049).

### Immunohistochemistry and interpretation

The DEF6 monoclonal antibody (clone 1 F2; Abnova, Taiwan; 1: 3000), p53 (clone: DO-7; Ventena; prediluted), and p16 (clone: E6H4; Ventena; prediluted) were used. Formalin-fixed and paraffin-embedded tissue sections were cut, deparaffinised and rehydrated. Autoclaved retrieval technique by using 10 mM citric acid buffer (10–20 min) and inhibited by endogenous peroxidase activity (0.3 % H_2_O_2_; 5 min) were conducted. Tissue sections were incubated with primary antibodies in an automated stainer system (Ventana, BenchMark XT). Tissue sections were then incubated with secondary antibody (dilution rate 1:100, 30 min), peroxidase-conjugated streptavidin (100 μg/mL), and 0.02 % 3,3’-diaminobenzidine tetrahydrochloride (DAB) (0.05 M Tris-HCl buffer with 0.03 % H_2_O_2_). All slides were counterstained with hematoxylin and analyzed by two pathologists (P-L Liew and C-L Chen).

The DEF6 cytoplasmic staining results were scored according to the percentages of positive cells: score 0 (less than 5 %), score 1 (5-24 %), score 2 (25-75 %), and score 3 (more than 75 %). The stromal lymphocytes were served as positive internal control. The results of p53 and p16 immunostains were scored according to the percentages of positive cells: score 0 (0 %), score 1 (1-24 %), score 2 (25-75 %), and score 3 (more than 75 %). Score 0 and score 3 of p53 were recorded as aberrant expression; whereas score 1 and score 2 of p53 were designated as insignificant expression.

### Cell lines and cell lysates

Total cell lysates from six ovarian carcinoma cells, including A2780, ES-2, TOV-21G, TOV-112D, OVCAR3, and HBT75, were used for the detection of DEF6 expression by Western blotting. A2780 (ovarian carcinoma cell line with unknown disease type identification) cell was cultured in RPMI-1640 with 10 % fetal bovine serum (FBS). ES-2 (clear cell carcinoma), TOV-21G (clear cell carcinoma) and TOV-112D (endometrioid carcinoma) were obtained from ATCC bioresource center (Manassas, VA, USA). ES-2 was cultured in McCoy’s 5 medium with 10 % FBS. TOV-21G and TOV-112D were cultured in a 50:50 mixture of Medium 199 and MCDB105 with 10 % FBS. Cells were grown to near confluent, washed with PBS, and then lysed by RIPA lysis buffer. The lysates of OVCAR3 (serous carcinoma) and HBT75 (serous carcinoma) cells were kindly provided by Prof. Chao-Lien Liu at the Department of Medical Laboratory Science and Biotechnology, Taipei Medical University.

### Western blot assay

Lysates from the six ovarian carcinoma cell lines and two positive control oral carcinoma cells, HSC-3 and SCC25, were separated in a 10 % polyacrylamide gel and transferred onto a PVDF membrane. After blocking, the blot was incubated with indicated antibodies overnight at 4 °C. Antibodies against DEF6and β-actin (Genetex, Irvine, CA, USA) were used as the primary antibodies. After washing, the blot was incubated with horseradish peroxidase-labeled goat anti-mouse/rabbit IgG (Jackson Laboratory). The expression profile of the proteins was visualized using a Western Lightening-ECL kit (PerkinElmer, Waltham, MA, USA).

### Statistical analyses

We performed statistical analyses by using SPSS for Windows software (SPSS, Chicago, IL). Data were expressed as median (interquartile range), mean (SD), and percentages. Chi-square test, Mann-Whitney U tests, and Student’s *t*-test were used as statistical methods. Spearman rank correlation and multivariate linear regressions with stepwise variable selection were performed to assess the significant associations between ordinal or continuous predictor variables. The Kaplan-Meier method, Cox proportional hazard regression model and multifactorial Cox regression analysis were used to examine all factors found to be prognostic of survival in univariate analysis and the analysis of DFS and OS. A *P*-value of less than 0.05 was considered statistically significance.

## Results

### Comparison of DEF6, p53 and p16 expressions in different histological subtypes

In our cohort of study, we collected 180 cases of ovarian carcinomas (75 high-grade serous, 41 clear cell, 36 mucinous, and 28 endometrioid carcinomas). We did not enroll cases of low-grade serous carcinoma because only three cases of low-grade serous carcinoma were diagnosed. The patients’ ages ranged from 25 to 93 years (mean: 53.8 years). Totally 109 early stage cases (FIGO stage I-II) and 71 advanced stage (FIGO stage III-IV), with a median follow-up time of 33.0 months (mean, 48.3 months; range, 1 to 146 months) were enrolled. The surgical procedures included total hysterectomy, bilateral salpingo-oophorectomy, pelvic and/or para-aortic lymph nodes sampling and omentectomy.

**Fig. 1:**
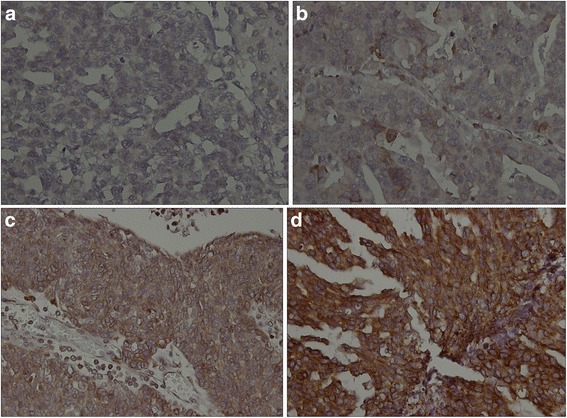
Cytoplasmic immunoexpression of DEF6 in high-grade serous carcinoma. Case representatives show (**a**) negative (less than 5 %): score 0; (**b**) weak (5–25 %): score 1; (**c**) moderate (26–75 %): score 2; (**d**) strong (more than 75 %): score 3 of tumor cells

As shown in Table 1, immunoexpressions of DEF6, p53 and p16 showed significant correlation with different histological subtypes of ovarian carcinoma. Score 3 immunoexpression of DEF6 could be assessed in 52 % (39/75) and 64.3 % (18/28) of high-grade serous carcinoma (Figs. 1a, b, c and d) and endometrioid carcinoma, respectively. Aberrant p53 expression was also observed in 82.7 % of high-grade serous carcinoma. The p16 expression was either cytoplasmic or showed a combination of nuclear and cytoplasmic immunoreactivity. The expression of p16 could be observed in 74.7 % (56/75) of high-grade serous carcinomas, and commonly lost (up to 80.6 %) in mucinous carcinomas. There was some variation in p16 staining in the endometrioid carcinoma and clear cell carcinoma groups. In summary, high-grade serous carcinoma showed the highest frequencies of increased DEF6 and p16 expressions, as well as aberrant p53 expression.

**Table 1:** Correlation of DEF6, p53 and p16 expression in histological subtypes of ovarian carcinoma

		Histology subtypes of ovarian carcinoma								
		Serous carcinoma (*N* = 75)		Mucinous carcinoma (*N* = 36)		Endometrioid carcinoma (*N* = 28)		Clear cell carcinoma (*N* = 41)		
Antibody	Score	No.	(%)	No.	(%)	No.	(%)	No.	(%)	*P*-value
DEF6	0	8	(10.7)	8	(22.2)	2	(7.1)	6	(14.6)	<0.001*
	1	7	(9.3)	12	(33.3)	4	(14.3)	15	(36.6)	
	2	21	(28.0)	9	(25.0)	4	(14.3)	11	(26.8)	
	3	39	(52.0)	7	(19.4)	18	(64.3)	9	(22.0)	
	NA	0	(0.0)	0	(0.0)	0	(0.0)	0	(0.0)	
p53	0	26	(34.7)	12	(33.3)	6	(21.4)	6	(14.6)	<0.001*
	1	11	(14.7)	17	(47.2)	18	(64.3)	29	(70.7)	
	2	1	(1.3)	2	(5.6)	1	(3.6)	2	(4.9)	
	3	36	(48.0)	5	(13.9)	3	(10.7)	4	(9.8)	
	NA	1	(1.3)	0	(0.0)	0	(0.0)	0	(0.0)	
p16	0	12	(16.0)	29	(80.6)	11	(39.3)	14	(34.1)	<0.001*
	1	6	(8.0)	3	(8.3)	6	(21.4)	16	(39.0)	
	2	0	(0.0)	0	(0.0)	0	(0.0)	0	(0.0)	
	3	56	(74.7)	3	(8.3)	11	(39.3)	10	(24.4)	
	NA	1	(1.3)	1	(2.8)	0	(0.0)	1	(2.4)	

NA, Not applicable (not enough material for analysis or technical limitations)

*P*- value Fisher’s exact or Chi-square test analysis (*significant difference)

### Expression of DEF6 in ovarian carcinoma cell lines

**Fig. 2:**
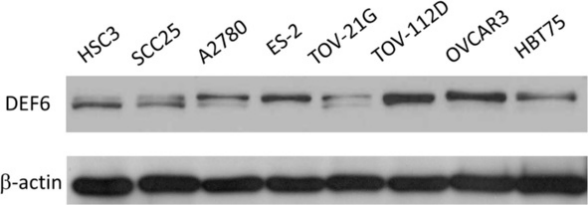
Expression of DEF6 in ovarian carcinoma cell lines. The expression profile of DEF6 was studied in six ovarian carcinoma cell lines. A2780: unknown type; ES-2 and TOV-21G: clear cell carcinoma; TOV-112D: endometrioid carcinoma; OVCAR3 and HBT75: serous carcinoma. Two oral carcinoma cells, HSC-3 and SCC25, were included as positive controls. β-actin is detected as a loading control

The expression profile of DEF6 was studied in several ovarian carcinoma cell lines by Western blot. Among six ovarian carcinoma cells examined, the expression of DEF6 in TOV-112D (endometrioid carcinoma) and OVCAR3 (serous carcinoma) cells were found to be relatively high when compared to other ovarian cells (Fig. 2). These findings were compatible with immunohistochemical results. This indicated that DEF6 was preferentially overexpressed in ovarian serous and endometrioid carcinomas. The level of DEF6 in A2780 (unknown histology subtype) and TOV-21G (clear cell carcinoma) was comparatively low among the six cells, however, they revealed a double-band pattern on the DEF6 region as similar to the oral carcinoma cells.

### Prognostic values of co-expression DEF6/p16 and DEF6/p53

We analyzed the prognostic significance of DEF6, p16 and p53 in the whole group of 180 cases of ovarian carcinoma. Four subgroups of patients each were formed according to DEF6/p16 and DEF6/p53 co-expressions (Table 2). The group with positive DEF6 and positive p16 expression was associated with the lowest OS (*P* = 0.027) and DFS (*P* = 0.023), whereas the DEF6 and p16 negative group had the highest OS and DFS. Similarly, the presence of aberrant p53 expression with concomitant DEF6 expression was statistically associated with worst prognosis, whereas the group negative for both had the best OS and DFS (*P* = 0.031 for OS and *P* = 0.028 for DFS, respectively).

**Table 2:** Overall survival (OS) and disease-free survival (DFS) according to DEF6/p16 and DEF6/p53 co-expressions

	OS (SE)	95 % CI	Median (SE) (95 % CI)	*P*-value	DFS (SE)	95 % CI	Median (SE) (95 % CI)	*P*-value
DEF6/p16 co-expression								
DEF6-negative/ p16-negative	75 (11)	53–98	90 (30) 30–149	0.027*	75 (11)	52–98	90 (30) 30–149	0.023*
DEF6-negative/ p16-positive	54 (16)	22–86	48 (30) 0–107		54 (16)	22–86	48 (30) 0–107	
DEF6-postive/ p16-negative	41 (10)	22–61	27 (10) 6–47		39 (9)	19–58	27 (9) 8–45	
DEF6-positive/ p16-positive	32 (6)	19–45	21 (4) 12–30		32 (6)	19–44	19 (3) 12–25	
DEF6/p53 co-expression								
DEF6-negative /p53 (scores 1 and 2)	73 (10)	52-93	72 (20) 31-113	0.031*	73 (10)	52-93	72 (20) 31-113	0.028*
DEF6-negative /p53 (scores 0 and 3)	64 (17)	30–99	76 (79) 0–230		64 (17)	30–97	76 (79) 0–230	
DEF6-postive /p53 (scores 1 and 2)	48 (11)	26–70	48 (25) 0–97		44 (10)	22–65	31 (16) 0–62	
DEF6-positive /p53 (scores 0 and 3)	29 (6)	16–41	19 (2) 13–24		28 (6)	16–41	17 (1) 14–19	

SE, Standard error; CI, confidence interval

*significant difference

Log-rank test

DEF6 positive: Scores 2 and 3; DEF6 negative: Scores 0 and 1

p16 positive: Scores 2 and 3; p16 negative: Scores 0 and 1

### Effects of DEF6, p16 and p53 expression on the patient’s overall survivals

**Fig. 3:**
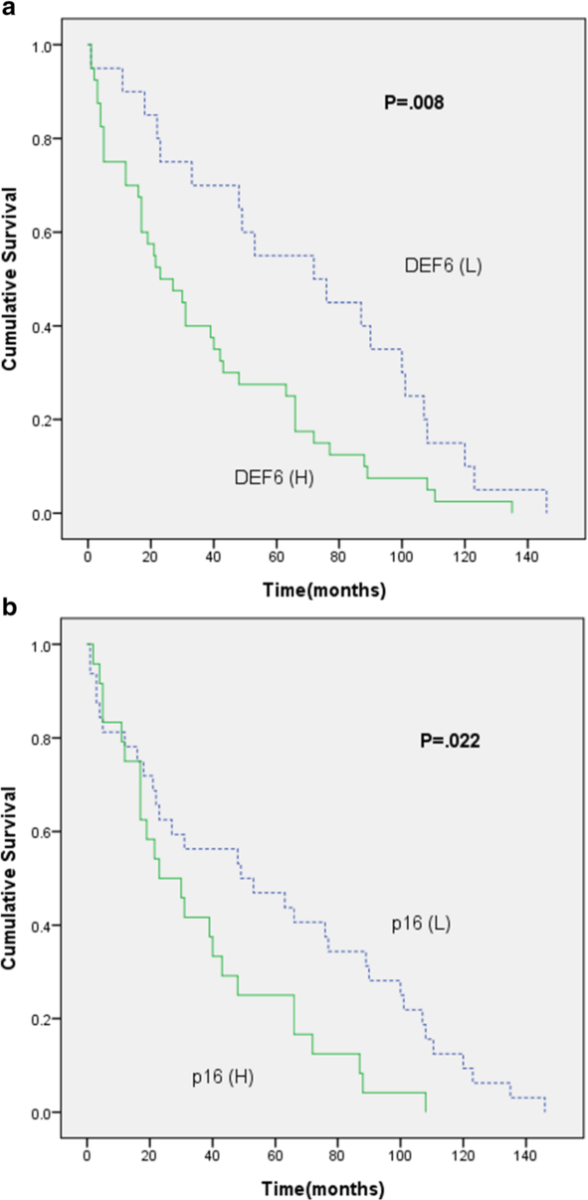
Kaplan-Meier curves for overall survival according to protein expression levels in ovarian carcinomas (*n* = 180). **a** Patients with high DEF6 expression (scores 2 and 3; *n* = 118) versus low DEF6 expression (scores 0 and 1; *n* = 62). (H: high DEF6 expression; L: low DEF6 expression); (**b**) Patients with high p16 expression (scores 2 and 3; *n* = 80) versus low p16 expression (scores 0 and 1; *n* = 97). (H: high p16 expression; L: low p16 expression)

**Fig. 4:**
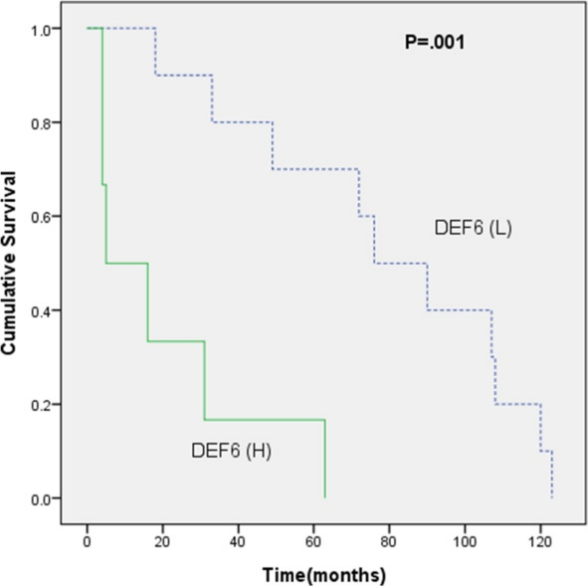
Kaplan-Meier curves for overall survival in patients with high DEF6 expression (scores 2 and 3; *n* = 21) versus low DEF6 expression (scores 0 and 1; *n* = 20) in clear cell carcinoma. (H: high DEF6 expression; L: low DEF6 expression)

Ovarian carcinoma patients with high DEF6 expression were associated with a poor overall survival compared with the patients with low DEF6 expression (*P* = 0.008; Fig. 3a). Also, ovarian carcinoma patients with high 16 expression had lower overall survival rate than those with low p16 expression, as determined using the Kaplan-Meier method (*P* = 0.022; Fig. 3b). Subsequently, we carried out survival analyses according to IHC evaluations in four different histological subtypes of ovarian carcinoma (see Additional file 1). Patients with aberrant p53 expression had shorter survival rate than those with insignificant p53 expression in high-grade serous carcinoma (*P* = 0.012). More importantly, in ovarian clear cell carcinoma, high expression of DEF6 was associated with shorter overall survival as compared to low DEF6 expression (*P* = 0.001, Fig. 4).

### Prognostic effects of clinicopathological factors and immunohistochemistry

Univariate (Table 3) and multivariate (Table 4) analyses were conducted to analyze the clinicopathological and IHC characteristics of the ovarian cancer patient cohorts to elucidate the crucial prognostic factors. Univariate analysis identified FIGO stage (*P* = 0.023 for OS and *P* = 0.034 for DFS), DEF6 (*P* = 0.013 for OS and *P* = 0.009 for DFS) and p16 (*P* = 0.026 for OS and *P* = 0.031 for DFS) as prognostic factors. Upon multivariate analysis, strong DEF6 was the only independent prognostic factor correlated with shorted OS (HR 2.115; *P* = 0.025) and DFS (HR 2.248; *P* = 0.016).

**Table 3:** Univariate analyses showing HRs for patient OS and DFS conferred age, FIGO stage, histologic subtypes, DEF6, p53, p16 and ER expression (*N* = 180)

Variables		Total No.	Overall survival (OS)			Disease-free survival (DFS)		
			HR	95 % CI	*P* -value	HR	95 % CI	*P* -value
Age, years					0.990			0.900
≦50		72	1.00^a^			1.00^a^		
>50		108	1.003	0.595–1.693		1.034	0.613–1.745	
FIGO stage					0.023*			0.034*
I		86	1.00^a^			1.00^a^		
II		23	1.141	0.518–2.511		1.119	0.509–2.460	
III		64	2.282	1.217–4.279		2.166	1.161–4.040	
IV		7	5.300	1.173–23.950		4.939	1.100–22.172	
Histologic subtypes					0.303			0.284
Serous carcinoma		75	1.00^a^			1.00^a^		
Mucinous carcinoma		36	0.529	0.247–1.135		0.531	0.247–1.138	
Endometrioid carcinoma		28	0.786	0.342–1.808		0.882	0.382–2.037	
Clear cell carcinoma		41	0.607	0.317–1.162		0.608	0.318–1.163	
DEF6					0.013*			0.009*
Scores 0 and 1		62	1.00^a^			1.00^a^		
Scores 2 and 3		118	2.041	1.159–3.594		2.119	1.202–3.737	
p16					0.026*			0.031*
Scores 0 and 1		97	1.00^a^			1.00^a^		
Scores 2 and 3		80	1.919	1.082–3.402		1.873	1.057–3.318	
p53					0.181			0.205
Scores 1 and 2		81	1.00^a^			1.00^a^		
Scores 0 and 3		98	1.450	0.841–2.499		1.421	0.825–2.448	

^a^Reference category for HR (Hazard Ratio) calculation with variable

CI: Confidence Interval. (*Significant difference)

**Table 4:** Multivariate analyses showing HRs for patient OS and DFS conferred FIGO stage, DEF6 and p16 expression (*N* = 180)

Variable		Overall survival (OS)			Disease-free survival (DFS)		
		HR	95 % CI	*P* -value	HR	95 % CI	*P* -value
FIGO stage							
I		1.00^a^			1.00^a^		
II		0.624	0.235–1.658	0.344	0.591	0.222–1.575	0.293
III		1.480	0.563–3.890	0.426	1.373	0.522–3.609	0.520
IV		3.845	0.734–20.133	0.111	3.496	0.671–18.206	0.137
DEF6				0.025*			0.016*
Score 0 and 1		1.00^a^			1.00^a^		
Score 2 and 3		2.115	1.097–4.079		2.248	1.161–4.352	
p16				0.611			0.611
Score 0, 1 and 2		1.00^a^			1.00^a^		
Score 3		1.250	0.530–2.945		1.250	0.529–2.953	

^a^Reference category for HR (Hazard Ratio) calculation with variable

CI: Confidence Interval. (*Significant difference)

## Discussion

DEF6 is a conserved protein associated with the functions of lymphocytes and is highly expressed in T-cells and T-cell homing organs [4–27]. DEF6 was expressed in breast cancer cells [20], oral squamous cell carcinoma [21], colorectal carcinoma [19], and in tumor vessels of renal cell carcinoma [22]. The role of DEF6 expression in human cancer is unclear, and the prognostic significance of DEF6 expression and the co-expression of p16 and p53 in ovarian carcinomas are largely unknown.

In this study of 180 cases of ovarian carcinoma using immunohistochemistry, a high expression of DEF6 was commonly found in ovarian carcinomas, and associated with different histology subtypes, advanced FIGO stage, and reduced overall survival (OS) and disease free survival (DFS). Strong expression of DEF6 was observed in high-grade serous carcinoma and endometrioid carcinoma. These findings were supported by the high expression of DEF6 in two serous, two clear cell and one endometrioid carcinoma cell lines, suggestive of the frequent expression of DEF6 in ovarian carcinoma tissues and cells.

There are many molecular markers possessing prognostic value. We compared the clinicopathological parameters and prognostic factors of DEF6, p16 and p53. The p16 is a cyclin-dependent kinase inhibitor which is integral to the retinoblastoma (Rb) gene-mediated control of the G_1_-S phase transition of the cell cycle [28]. It was also shown that ectopic expression of DEF6 shortened the G_1_ interval in the cell cycle, and increased cyclin D1 expression [21]. It is therefore important to explore the cooperation between the two genes in the tumor progression. Although p16 has been widely regarded as a surrogate marker of high-risk human papillomavirus (HPV) in uterine cervical pathology, the role of p16 protein expression in ovarian carcinomas remains limited. In our study (Table 1), we found the highest percentage of score 3 p16 expression (74.7 %) in high-grade serous carcinoma, an ovarian carcinoma with notorious poor outcome, and similarly DEF6 (52 %). Our findings suggest that p16 is also a valuable prognostic marker for ovarian carcinomas. Recent studies have investigated the diagnostic role of p16 and prognostic indicator of p16 with clinical outcome in ovarian/tubal high-grade serous carcinoma. As p16 was expressed in the majority of p53-positive and p53-negative serous tubal intraepithelial carcinomas, the addition of p16 helped to compensate the practical limitations of p53 in the diagnosis of serous tubal intraepithelial carcinomas [24]. Moreover, a recent study on ovarian/tubal high grade serous carcinomas revealed three distinct subgroups according to p16 expression and RB1 status (i.e., p16 homogenous stain/RB1-, p16 homogenous stain/RB1+, and p16 heterogeneous stain/RB1+), which possessed clinical relevance for stage and patient outcome upon multivariate analysis [29]. We also assessed the prognostic significance of the status of DEF6/p16 co-expression. Notably, the co-expression of both proteins was associated closely with adverse clinical outcome (Table 2). The OS and DFS, in particular, were the shortest in the group with strong DEF6 and p16 co-expression, followed by DEF6+/p16- and DEF6-/p16+, while the DEF6-/p16- group had the longest survival rates (*P* = 0.027 for OS and *P* = 0.023 for DFS, respectively). These findings suggest that DEF6 and p16 positively interact and contribute to the tumor progression in ovarian carcinoma, and the molecular mechanisms deserve to be further studied. This suggestion is supported by the observations that both proteins play important role in the control of the G_1_-S phase transition of the cell cycle [21, 28].

DEF6 is a novel p53 target gene and negatively regulated by p53, it can suppress cisplatin-mediated apoptosis of breast cancer cells [23]. In the present study, we found that strong DEF6 expression and aberrant p53 expression had the shortest survival, whereas co-expression of insignificant p53 expression and negative DEF6 displayed the longest survival rate, with the other two groups in between (*P* = 0.031 for OS and *P* = 0.028 for DFS, Table 3). Thus, the evaluation of the p53 status coupled with DEF6 might be important for risk stratification, which certainly awaits sequencing validation of the p53 status for further clarification.

We further evaluated the overall survival rates of DEF6, p16 and p53 in all patients and four different histology subtypes of ovarian carcinoma by using Kaplan-Meier analysis. Significantly shorter survival rates were seen in patients with high expressions of DEF6 (*P* = 0.008) and p16 (*P* = 0.022). Patients with aberrant p53 expression in high-grade serous carcinoma had shorter overall survival (*P* = 0.012). Surprisingly, high expression of DEF6 in clear cell carcinoma was significantly correlated to shorter overall survival (*P* = 0.001). We enrolled in total 41 cases of ovarian clear cell carcinoma (30 FIGO stage I, 4 FIGO stage II and 7 FIGO stage III) in this study. Therefore, our study suggests that patients of ovarian clear cell carcinoma with high DEF6 expression deserve a poor prognostic factor compared with other histological subtypes. Our study is the first report on the prognostic impact of DEF6 overexpression in early-stage ovarian clear cell carcinomas, however in-depth investigations are necessary.

Univariate analysis showed that FIGO stage, DEF6 and p16 were associated with shorter survival (Table 3). Upon multivariate analysis, DEF6 remained the significant prognostic value (Table 4). These findings suggest that DEF6 in ovarian carcinomas may facilitate tumor cell growth or proliferation, motility, invasion and metastasis, leading to high tumor stage and hence poor prognosis.

## Conclusions

DEF6 was often overexpressed in ovarian carcinomas, particularly in high-grade serous carcinoma and endometrioid carcinoma cells and tissues. DEF6 overexpression in early-stage ovarian clear cell carcinomas may have potential role as a poor prognostic factor. Multivariate analysis showed that DEF6 might serve as independent prognostic biomarker. Our findings suggest that DEF6 contribute cooperatively with p16 and p53, another two important cell cycle regulators, toward high tumor stage and poor OS and DFS in ovarian carcinomas. Hence DEF6 expression deserves further investigation especially its potential as therapeutic target in the dreadful ovarian carcinomas.

## Abbreviations

DFS, disease-free survival; FIGO, International Federation of Gynecology and Obstetrics; H&E, hematoxylin and eosin; HR, Hazard Ratio; IHC, immunohistochemical; IBP, interferon regulatory factor 4 binding protein; OS, overall survival; TMA, tissue microarray

## Additional file

Additional file 1: Figure S1. Kaplan-Meier curves for overall survival in four histological subtypes of ovarian carcinoma patients according to DEF6, p16 and p53 expressions. A, Patients with high DEF6 expression (scores 2 and 3; *n* = 60) versus low DEF6 expression (scores 0 and 1; *n* = 15) in high-grade serous carcinoma. (H: high DEF6 expression; L: low DEF6 expression). B, Patients with high p16 expression (scores 2 and 3; *n* = 56) versus low p16 expression (scores 0 and 1; *n* = 18) in high-grade serous carcinoma. (H: high p16 expression; L: low p16 expression). C, Patients with aberrant p53 expression (scores 0 and 3; *n* = 62) versus insignificant p53 expression (scores 1 and 2; *n* = 12) in high-grade serous carcinoma. D, Patients with high DEF6 expression (scores 2 and 3; *n* = 20) versus low DEF6 expression (scores 0 and 1; *n* = 16) in mucinous carcinoma. (H: high DEF6 expression; L: low DEF6 expression). E, Patients with high p16 expression (scores 2 and 3; *n* = 32) versus low p16 expression (scores 0 and 1; *n* = 3) in mucinous carcinoma. (H: high p16 expression; L: low p16 expression). F, Patients with aberrant p53 expression (scores 0 and 3; *n* = 17) versus insignificant p53 expression (scores 1 and 2; *n* = 19) in mucinous carcinoma. G, Patients with high DEF6 expression (scores 2 and 3; *n* = 6) versus low DEF6 expression (scores 0 and 1; *n* = 22) in endometrioid carcinoma. (H: high DEF6 expression; L: low DEF6 expression). H, Patients with high p16 expression (scores 2 and 3; *n* = 17) versus low p16 expression (scores 0 and 1; *n* = 11) in endometrioid carcinoma. (H: high p16 expression; L: low p16 expression). I, Patients with aberrant p53 expression (scores 0 and 3; *n* = 9) versus insignificant p53 expression (scores 1 and 2; *n* = 19) in endometrioid carcinoma. J, Patients with high p16 expression (scores 2 and 3; *n* = 30) versus low p16 expression (scores 0 and 1; *n* = 11) in clear cell carcinoma. (H: high p16 expression; L: low p16 expression). K, Patients with aberrant p53 expression (scores 0 and 3; *n* = 10) versus insignificant p53 expression (scores 1 and 2; *n* = 31) in clear cell carcinoma. (ZIP 195 kb)
