# Supplementary figures and images for: DEF6 expression in ovarian carcinoma correlates with poor patient survival
Source: Diagn Pathol. 2016 Aug 3;11:68. doi: 10.1186/s13000-016-0518-y (PMC4973116; doi:10.1186/s13000-016-0518-y)

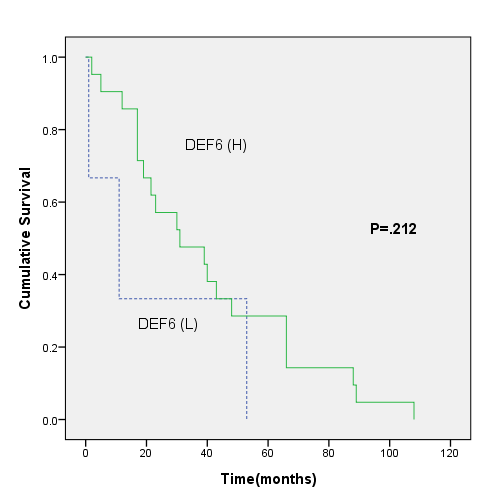

Supplement: Additional file 1: Figure S1. — Kaplan-Meier curves for overall survival in four histological subtypes of ovarian carcinoma patients according to DEF6, p16 and p53 expressions. A, Patients with high DEF6 expression (scores 2 and 3; n = 60) versus low DEF6 expression (scores 0 and 1; n = 15) in high-grade serous carcinoma. (H: high DEF6 expression; L: low DEF6 expression). B, Patients with high p16 expression (scores 2 and 3; n = 56) versus low p16 expression (scores 0 and 1; n = 18) in high-grade serous carcinoma. (H: high p16 expression; L: low p16 expression). C, Patients with aberrant p53 expression (scores 0 and 3; n = 62) versus insignificant p53 expression (scores 1 and 2; n = 12) in high-grade serous carcinoma. D, Patients with high DEF6 expression (scores 2 and 3; n = 20) versus low DEF6 expression (scores 0 and 1; n = 16) in mucinous carcinoma. (H: high DEF6 expression; L: low DEF6 expression). E, Patients with high p16 expression (scores 2 and 3; n = 32) versus low p16 expression (scores 0 and 1; n = 3) in mucinous carcinoma. (H: high p16 expression; L: low p16 expression). F, Patients with aberrant p53 expression (scores 0 and 3; n = 17) versus insignificant p53 expression (scores 1 and 2; n = 19) in mucinous carcinoma. G, Patients with high DEF6 expression (scores 2 and 3; n = 6) versus low DEF6 expression (scores 0 and 1; n = 22) in endometrioid carcinoma. (H: high DEF6 expression; L: low DEF6 expression). H, Patients with high p16 expression (scores 2 and 3; n = 17) versus low p16 expression (scores 0 and 1; n = 11) in endometrioid carcinoma. (H: high p16 expression; L: low p16 expression). I, Patients with aberrant p53 expression (scores 0 and 3; n = 9) versus insignificant p53 expression (scores 1 and 2; n = 19) in endometrioid carcinoma. J, Patients with high p16 expression (scores 2 and 3; n = 30) versus low p16 expression (scores 0 and 1; n = 11) in clear cell carcinoma. (H: high p16 expression; L: low p16 expression). K, Patients with aberrant p53 expression (scores 0 and 3; n [file 13000_2016_518_MOESM1_ESM.zip › Figure_Supp1A.tif]

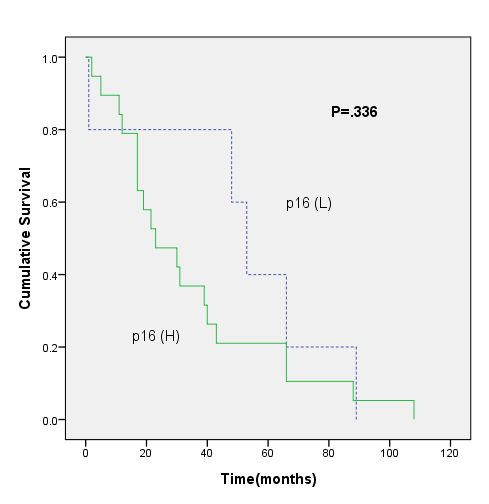

Supplement: Additional file 1: Figure S1. — Kaplan-Meier curves for overall survival in four histological subtypes of ovarian carcinoma patients according to DEF6, p16 and p53 expressions. A, Patients with high DEF6 expression (scores 2 and 3; n = 60) versus low DEF6 expression (scores 0 and 1; n = 15) in high-grade serous carcinoma. (H: high DEF6 expression; L: low DEF6 expression). B, Patients with high p16 expression (scores 2 and 3; n = 56) versus low p16 expression (scores 0 and 1; n = 18) in high-grade serous carcinoma. (H: high p16 expression; L: low p16 expression). C, Patients with aberrant p53 expression (scores 0 and 3; n = 62) versus insignificant p53 expression (scores 1 and 2; n = 12) in high-grade serous carcinoma. D, Patients with high DEF6 expression (scores 2 and 3; n = 20) versus low DEF6 expression (scores 0 and 1; n = 16) in mucinous carcinoma. (H: high DEF6 expression; L: low DEF6 expression). E, Patients with high p16 expression (scores 2 and 3; n = 32) versus low p16 expression (scores 0 and 1; n = 3) in mucinous carcinoma. (H: high p16 expression; L: low p16 expression). F, Patients with aberrant p53 expression (scores 0 and 3; n = 17) versus insignificant p53 expression (scores 1 and 2; n = 19) in mucinous carcinoma. G, Patients with high DEF6 expression (scores 2 and 3; n = 6) versus low DEF6 expression (scores 0 and 1; n = 22) in endometrioid carcinoma. (H: high DEF6 expression; L: low DEF6 expression). H, Patients with high p16 expression (scores 2 and 3; n = 17) versus low p16 expression (scores 0 and 1; n = 11) in endometrioid carcinoma. (H: high p16 expression; L: low p16 expression). I, Patients with aberrant p53 expression (scores 0 and 3; n = 9) versus insignificant p53 expression (scores 1 and 2; n = 19) in endometrioid carcinoma. J, Patients with high p16 expression (scores 2 and 3; n = 30) versus low p16 expression (scores 0 and 1; n = 11) in clear cell carcinoma. (H: high p16 expression; L: low p16 expression). K, Patients with aberrant p53 expression (scores 0 and 3; n [file 13000_2016_518_MOESM1_ESM.zip › Figure_Supp1B.tif]

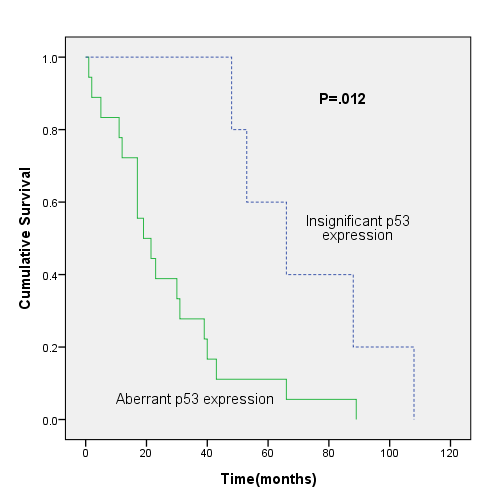

Supplement: Additional file 1: Figure S1. — Kaplan-Meier curves for overall survival in four histological subtypes of ovarian carcinoma patients according to DEF6, p16 and p53 expressions. A, Patients with high DEF6 expression (scores 2 and 3; n = 60) versus low DEF6 expression (scores 0 and 1; n = 15) in high-grade serous carcinoma. (H: high DEF6 expression; L: low DEF6 expression). B, Patients with high p16 expression (scores 2 and 3; n = 56) versus low p16 expression (scores 0 and 1; n = 18) in high-grade serous carcinoma. (H: high p16 expression; L: low p16 expression). C, Patients with aberrant p53 expression (scores 0 and 3; n = 62) versus insignificant p53 expression (scores 1 and 2; n = 12) in high-grade serous carcinoma. D, Patients with high DEF6 expression (scores 2 and 3; n = 20) versus low DEF6 expression (scores 0 and 1; n = 16) in mucinous carcinoma. (H: high DEF6 expression; L: low DEF6 expression). E, Patients with high p16 expression (scores 2 and 3; n = 32) versus low p16 expression (scores 0 and 1; n = 3) in mucinous carcinoma. (H: high p16 expression; L: low p16 expression). F, Patients with aberrant p53 expression (scores 0 and 3; n = 17) versus insignificant p53 expression (scores 1 and 2; n = 19) in mucinous carcinoma. G, Patients with high DEF6 expression (scores 2 and 3; n = 6) versus low DEF6 expression (scores 0 and 1; n = 22) in endometrioid carcinoma. (H: high DEF6 expression; L: low DEF6 expression). H, Patients with high p16 expression (scores 2 and 3; n = 17) versus low p16 expression (scores 0 and 1; n = 11) in endometrioid carcinoma. (H: high p16 expression; L: low p16 expression). I, Patients with aberrant p53 expression (scores 0 and 3; n = 9) versus insignificant p53 expression (scores 1 and 2; n = 19) in endometrioid carcinoma. J, Patients with high p16 expression (scores 2 and 3; n = 30) versus low p16 expression (scores 0 and 1; n = 11) in clear cell carcinoma. (H: high p16 expression; L: low p16 expression). K, Patients with aberrant p53 expression (scores 0 and 3; n [file 13000_2016_518_MOESM1_ESM.zip › Figure_Supp1C.tif]

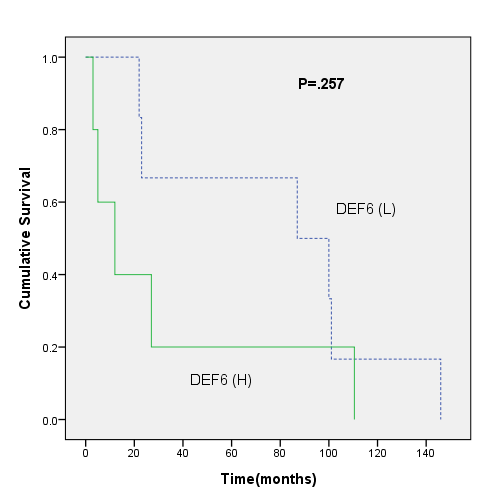

Supplement: Additional file 1: Figure S1. — Kaplan-Meier curves for overall survival in four histological subtypes of ovarian carcinoma patients according to DEF6, p16 and p53 expressions. A, Patients with high DEF6 expression (scores 2 and 3; n = 60) versus low DEF6 expression (scores 0 and 1; n = 15) in high-grade serous carcinoma. (H: high DEF6 expression; L: low DEF6 expression). B, Patients with high p16 expression (scores 2 and 3; n = 56) versus low p16 expression (scores 0 and 1; n = 18) in high-grade serous carcinoma. (H: high p16 expression; L: low p16 expression). C, Patients with aberrant p53 expression (scores 0 and 3; n = 62) versus insignificant p53 expression (scores 1 and 2; n = 12) in high-grade serous carcinoma. D, Patients with high DEF6 expression (scores 2 and 3; n = 20) versus low DEF6 expression (scores 0 and 1; n = 16) in mucinous carcinoma. (H: high DEF6 expression; L: low DEF6 expression). E, Patients with high p16 expression (scores 2 and 3; n = 32) versus low p16 expression (scores 0 and 1; n = 3) in mucinous carcinoma. (H: high p16 expression; L: low p16 expression). F, Patients with aberrant p53 expression (scores 0 and 3; n = 17) versus insignificant p53 expression (scores 1 and 2; n = 19) in mucinous carcinoma. G, Patients with high DEF6 expression (scores 2 and 3; n = 6) versus low DEF6 expression (scores 0 and 1; n = 22) in endometrioid carcinoma. (H: high DEF6 expression; L: low DEF6 expression). H, Patients with high p16 expression (scores 2 and 3; n = 17) versus low p16 expression (scores 0 and 1; n = 11) in endometrioid carcinoma. (H: high p16 expression; L: low p16 expression). I, Patients with aberrant p53 expression (scores 0 and 3; n = 9) versus insignificant p53 expression (scores 1 and 2; n = 19) in endometrioid carcinoma. J, Patients with high p16 expression (scores 2 and 3; n = 30) versus low p16 expression (scores 0 and 1; n = 11) in clear cell carcinoma. (H: high p16 expression; L: low p16 expression). K, Patients with aberrant p53 expression (scores 0 and 3; n [file 13000_2016_518_MOESM1_ESM.zip › FIgure_Supp1D.tif]

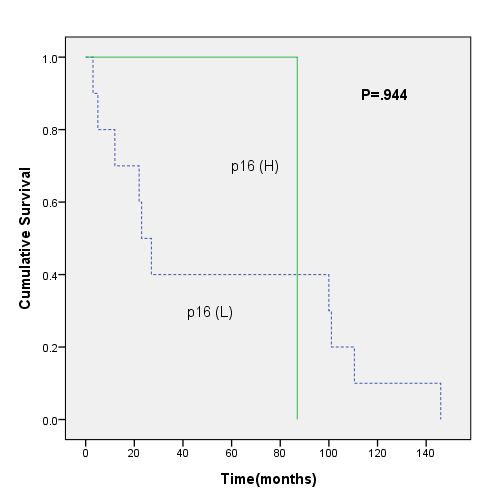

Supplement: Additional file 1: Figure S1. — Kaplan-Meier curves for overall survival in four histological subtypes of ovarian carcinoma patients according to DEF6, p16 and p53 expressions. A, Patients with high DEF6 expression (scores 2 and 3; n = 60) versus low DEF6 expression (scores 0 and 1; n = 15) in high-grade serous carcinoma. (H: high DEF6 expression; L: low DEF6 expression). B, Patients with high p16 expression (scores 2 and 3; n = 56) versus low p16 expression (scores 0 and 1; n = 18) in high-grade serous carcinoma. (H: high p16 expression; L: low p16 expression). C, Patients with aberrant p53 expression (scores 0 and 3; n = 62) versus insignificant p53 expression (scores 1 and 2; n = 12) in high-grade serous carcinoma. D, Patients with high DEF6 expression (scores 2 and 3; n = 20) versus low DEF6 expression (scores 0 and 1; n = 16) in mucinous carcinoma. (H: high DEF6 expression; L: low DEF6 expression). E, Patients with high p16 expression (scores 2 and 3; n = 32) versus low p16 expression (scores 0 and 1; n = 3) in mucinous carcinoma. (H: high p16 expression; L: low p16 expression). F, Patients with aberrant p53 expression (scores 0 and 3; n = 17) versus insignificant p53 expression (scores 1 and 2; n = 19) in mucinous carcinoma. G, Patients with high DEF6 expression (scores 2 and 3; n = 6) versus low DEF6 expression (scores 0 and 1; n = 22) in endometrioid carcinoma. (H: high DEF6 expression; L: low DEF6 expression). H, Patients with high p16 expression (scores 2 and 3; n = 17) versus low p16 expression (scores 0 and 1; n = 11) in endometrioid carcinoma. (H: high p16 expression; L: low p16 expression). I, Patients with aberrant p53 expression (scores 0 and 3; n = 9) versus insignificant p53 expression (scores 1 and 2; n = 19) in endometrioid carcinoma. J, Patients with high p16 expression (scores 2 and 3; n = 30) versus low p16 expression (scores 0 and 1; n = 11) in clear cell carcinoma. (H: high p16 expression; L: low p16 expression). K, Patients with aberrant p53 expression (scores 0 and 3; n [file 13000_2016_518_MOESM1_ESM.zip › FIgure_Supp1E.tif]

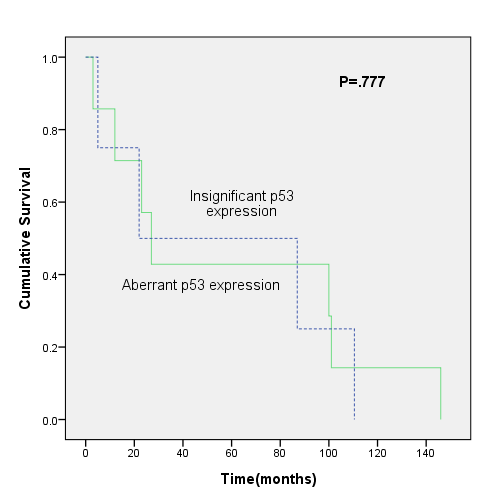

Supplement: Additional file 1: Figure S1. — Kaplan-Meier curves for overall survival in four histological subtypes of ovarian carcinoma patients according to DEF6, p16 and p53 expressions. A, Patients with high DEF6 expression (scores 2 and 3; n = 60) versus low DEF6 expression (scores 0 and 1; n = 15) in high-grade serous carcinoma. (H: high DEF6 expression; L: low DEF6 expression). B, Patients with high p16 expression (scores 2 and 3; n = 56) versus low p16 expression (scores 0 and 1; n = 18) in high-grade serous carcinoma. (H: high p16 expression; L: low p16 expression). C, Patients with aberrant p53 expression (scores 0 and 3; n = 62) versus insignificant p53 expression (scores 1 and 2; n = 12) in high-grade serous carcinoma. D, Patients with high DEF6 expression (scores 2 and 3; n = 20) versus low DEF6 expression (scores 0 and 1; n = 16) in mucinous carcinoma. (H: high DEF6 expression; L: low DEF6 expression). E, Patients with high p16 expression (scores 2 and 3; n = 32) versus low p16 expression (scores 0 and 1; n = 3) in mucinous carcinoma. (H: high p16 expression; L: low p16 expression). F, Patients with aberrant p53 expression (scores 0 and 3; n = 17) versus insignificant p53 expression (scores 1 and 2; n = 19) in mucinous carcinoma. G, Patients with high DEF6 expression (scores 2 and 3; n = 6) versus low DEF6 expression (scores 0 and 1; n = 22) in endometrioid carcinoma. (H: high DEF6 expression; L: low DEF6 expression). H, Patients with high p16 expression (scores 2 and 3; n = 17) versus low p16 expression (scores 0 and 1; n = 11) in endometrioid carcinoma. (H: high p16 expression; L: low p16 expression). I, Patients with aberrant p53 expression (scores 0 and 3; n = 9) versus insignificant p53 expression (scores 1 and 2; n = 19) in endometrioid carcinoma. J, Patients with high p16 expression (scores 2 and 3; n = 30) versus low p16 expression (scores 0 and 1; n = 11) in clear cell carcinoma. (H: high p16 expression; L: low p16 expression). K, Patients with aberrant p53 expression (scores 0 and 3; n [file 13000_2016_518_MOESM1_ESM.zip › Figure_Supp1F.tif]

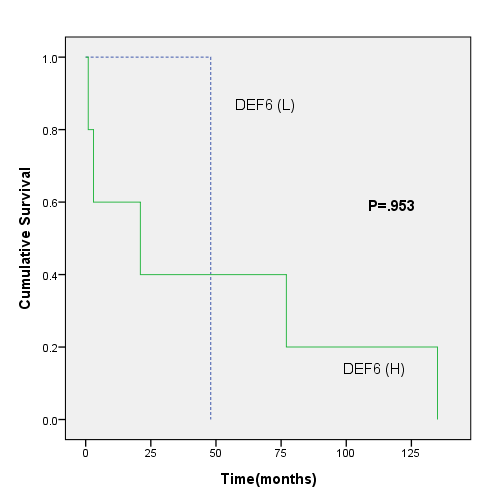

Supplement: Additional file 1: Figure S1. — Kaplan-Meier curves for overall survival in four histological subtypes of ovarian carcinoma patients according to DEF6, p16 and p53 expressions. A, Patients with high DEF6 expression (scores 2 and 3; n = 60) versus low DEF6 expression (scores 0 and 1; n = 15) in high-grade serous carcinoma. (H: high DEF6 expression; L: low DEF6 expression). B, Patients with high p16 expression (scores 2 and 3; n = 56) versus low p16 expression (scores 0 and 1; n = 18) in high-grade serous carcinoma. (H: high p16 expression; L: low p16 expression). C, Patients with aberrant p53 expression (scores 0 and 3; n = 62) versus insignificant p53 expression (scores 1 and 2; n = 12) in high-grade serous carcinoma. D, Patients with high DEF6 expression (scores 2 and 3; n = 20) versus low DEF6 expression (scores 0 and 1; n = 16) in mucinous carcinoma. (H: high DEF6 expression; L: low DEF6 expression). E, Patients with high p16 expression (scores 2 and 3; n = 32) versus low p16 expression (scores 0 and 1; n = 3) in mucinous carcinoma. (H: high p16 expression; L: low p16 expression). F, Patients with aberrant p53 expression (scores 0 and 3; n = 17) versus insignificant p53 expression (scores 1 and 2; n = 19) in mucinous carcinoma. G, Patients with high DEF6 expression (scores 2 and 3; n = 6) versus low DEF6 expression (scores 0 and 1; n = 22) in endometrioid carcinoma. (H: high DEF6 expression; L: low DEF6 expression). H, Patients with high p16 expression (scores 2 and 3; n = 17) versus low p16 expression (scores 0 and 1; n = 11) in endometrioid carcinoma. (H: high p16 expression; L: low p16 expression). I, Patients with aberrant p53 expression (scores 0 and 3; n = 9) versus insignificant p53 expression (scores 1 and 2; n = 19) in endometrioid carcinoma. J, Patients with high p16 expression (scores 2 and 3; n = 30) versus low p16 expression (scores 0 and 1; n = 11) in clear cell carcinoma. (H: high p16 expression; L: low p16 expression). K, Patients with aberrant p53 expression (scores 0 and 3; n [file 13000_2016_518_MOESM1_ESM.zip › FIgure_Supp1G.tif]

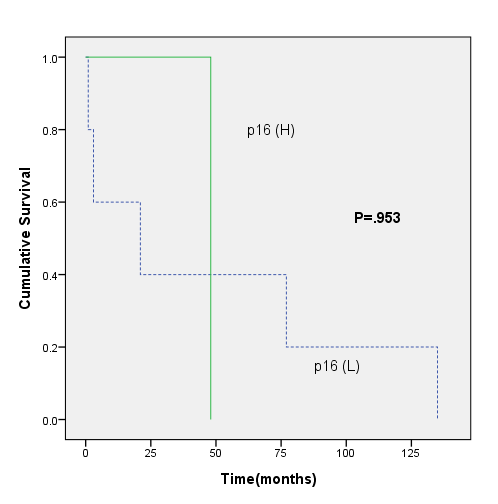

Supplement: Additional file 1: Figure S1. — Kaplan-Meier curves for overall survival in four histological subtypes of ovarian carcinoma patients according to DEF6, p16 and p53 expressions. A, Patients with high DEF6 expression (scores 2 and 3; n = 60) versus low DEF6 expression (scores 0 and 1; n = 15) in high-grade serous carcinoma. (H: high DEF6 expression; L: low DEF6 expression). B, Patients with high p16 expression (scores 2 and 3; n = 56) versus low p16 expression (scores 0 and 1; n = 18) in high-grade serous carcinoma. (H: high p16 expression; L: low p16 expression). C, Patients with aberrant p53 expression (scores 0 and 3; n = 62) versus insignificant p53 expression (scores 1 and 2; n = 12) in high-grade serous carcinoma. D, Patients with high DEF6 expression (scores 2 and 3; n = 20) versus low DEF6 expression (scores 0 and 1; n = 16) in mucinous carcinoma. (H: high DEF6 expression; L: low DEF6 expression). E, Patients with high p16 expression (scores 2 and 3; n = 32) versus low p16 expression (scores 0 and 1; n = 3) in mucinous carcinoma. (H: high p16 expression; L: low p16 expression). F, Patients with aberrant p53 expression (scores 0 and 3; n = 17) versus insignificant p53 expression (scores 1 and 2; n = 19) in mucinous carcinoma. G, Patients with high DEF6 expression (scores 2 and 3; n = 6) versus low DEF6 expression (scores 0 and 1; n = 22) in endometrioid carcinoma. (H: high DEF6 expression; L: low DEF6 expression). H, Patients with high p16 expression (scores 2 and 3; n = 17) versus low p16 expression (scores 0 and 1; n = 11) in endometrioid carcinoma. (H: high p16 expression; L: low p16 expression). I, Patients with aberrant p53 expression (scores 0 and 3; n = 9) versus insignificant p53 expression (scores 1 and 2; n = 19) in endometrioid carcinoma. J, Patients with high p16 expression (scores 2 and 3; n = 30) versus low p16 expression (scores 0 and 1; n = 11) in clear cell carcinoma. (H: high p16 expression; L: low p16 expression). K, Patients with aberrant p53 expression (scores 0 and 3; n [file 13000_2016_518_MOESM1_ESM.zip › Figure_Supp1H.tif]

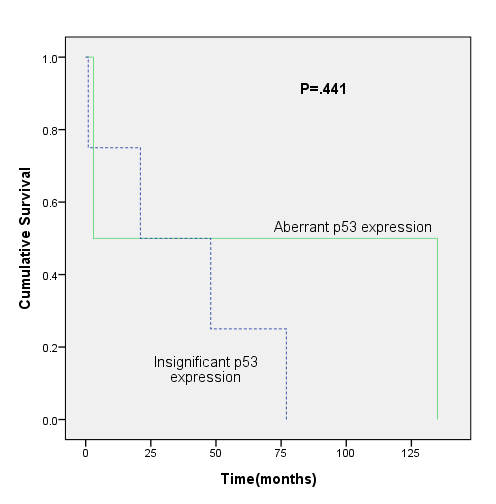

Supplement: Additional file 1: Figure S1. — Kaplan-Meier curves for overall survival in four histological subtypes of ovarian carcinoma patients according to DEF6, p16 and p53 expressions. A, Patients with high DEF6 expression (scores 2 and 3; n = 60) versus low DEF6 expression (scores 0 and 1; n = 15) in high-grade serous carcinoma. (H: high DEF6 expression; L: low DEF6 expression). B, Patients with high p16 expression (scores 2 and 3; n = 56) versus low p16 expression (scores 0 and 1; n = 18) in high-grade serous carcinoma. (H: high p16 expression; L: low p16 expression). C, Patients with aberrant p53 expression (scores 0 and 3; n = 62) versus insignificant p53 expression (scores 1 and 2; n = 12) in high-grade serous carcinoma. D, Patients with high DEF6 expression (scores 2 and 3; n = 20) versus low DEF6 expression (scores 0 and 1; n = 16) in mucinous carcinoma. (H: high DEF6 expression; L: low DEF6 expression). E, Patients with high p16 expression (scores 2 and 3; n = 32) versus low p16 expression (scores 0 and 1; n = 3) in mucinous carcinoma. (H: high p16 expression; L: low p16 expression). F, Patients with aberrant p53 expression (scores 0 and 3; n = 17) versus insignificant p53 expression (scores 1 and 2; n = 19) in mucinous carcinoma. G, Patients with high DEF6 expression (scores 2 and 3; n = 6) versus low DEF6 expression (scores 0 and 1; n = 22) in endometrioid carcinoma. (H: high DEF6 expression; L: low DEF6 expression). H, Patients with high p16 expression (scores 2 and 3; n = 17) versus low p16 expression (scores 0 and 1; n = 11) in endometrioid carcinoma. (H: high p16 expression; L: low p16 expression). I, Patients with aberrant p53 expression (scores 0 and 3; n = 9) versus insignificant p53 expression (scores 1 and 2; n = 19) in endometrioid carcinoma. J, Patients with high p16 expression (scores 2 and 3; n = 30) versus low p16 expression (scores 0 and 1; n = 11) in clear cell carcinoma. (H: high p16 expression; L: low p16 expression). K, Patients with aberrant p53 expression (scores 0 and 3; n [file 13000_2016_518_MOESM1_ESM.zip › FIgure_Supp1I.tif]

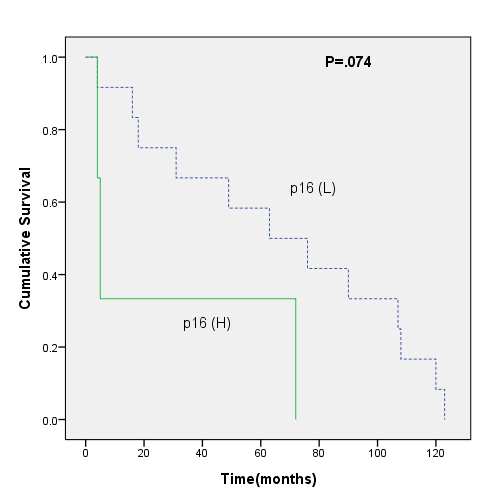

Supplement: Additional file 1: Figure S1. — Kaplan-Meier curves for overall survival in four histological subtypes of ovarian carcinoma patients according to DEF6, p16 and p53 expressions. A, Patients with high DEF6 expression (scores 2 and 3; n = 60) versus low DEF6 expression (scores 0 and 1; n = 15) in high-grade serous carcinoma. (H: high DEF6 expression; L: low DEF6 expression). B, Patients with high p16 expression (scores 2 and 3; n = 56) versus low p16 expression (scores 0 and 1; n = 18) in high-grade serous carcinoma. (H: high p16 expression; L: low p16 expression). C, Patients with aberrant p53 expression (scores 0 and 3; n = 62) versus insignificant p53 expression (scores 1 and 2; n = 12) in high-grade serous carcinoma. D, Patients with high DEF6 expression (scores 2 and 3; n = 20) versus low DEF6 expression (scores 0 and 1; n = 16) in mucinous carcinoma. (H: high DEF6 expression; L: low DEF6 expression). E, Patients with high p16 expression (scores 2 and 3; n = 32) versus low p16 expression (scores 0 and 1; n = 3) in mucinous carcinoma. (H: high p16 expression; L: low p16 expression). F, Patients with aberrant p53 expression (scores 0 and 3; n = 17) versus insignificant p53 expression (scores 1 and 2; n = 19) in mucinous carcinoma. G, Patients with high DEF6 expression (scores 2 and 3; n = 6) versus low DEF6 expression (scores 0 and 1; n = 22) in endometrioid carcinoma. (H: high DEF6 expression; L: low DEF6 expression). H, Patients with high p16 expression (scores 2 and 3; n = 17) versus low p16 expression (scores 0 and 1; n = 11) in endometrioid carcinoma. (H: high p16 expression; L: low p16 expression). I, Patients with aberrant p53 expression (scores 0 and 3; n = 9) versus insignificant p53 expression (scores 1 and 2; n = 19) in endometrioid carcinoma. J, Patients with high p16 expression (scores 2 and 3; n = 30) versus low p16 expression (scores 0 and 1; n = 11) in clear cell carcinoma. (H: high p16 expression; L: low p16 expression). K, Patients with aberrant p53 expression (scores 0 and 3; n [file 13000_2016_518_MOESM1_ESM.zip › Figure_Supp1J.tif]

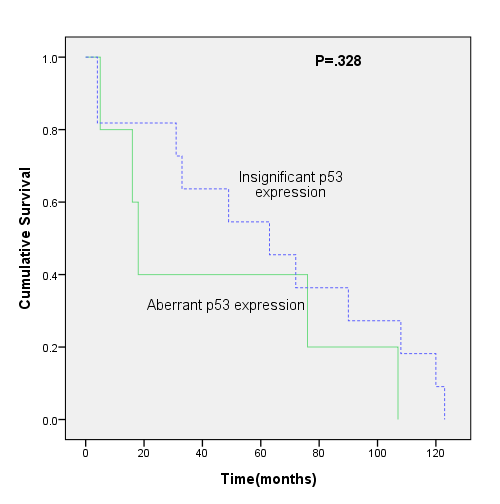

Supplement: Additional file 1: Figure S1. — Kaplan-Meier curves for overall survival in four histological subtypes of ovarian carcinoma patients according to DEF6, p16 and p53 expressions. A, Patients with high DEF6 expression (scores 2 and 3; n = 60) versus low DEF6 expression (scores 0 and 1; n = 15) in high-grade serous carcinoma. (H: high DEF6 expression; L: low DEF6 expression). B, Patients with high p16 expression (scores 2 and 3; n = 56) versus low p16 expression (scores 0 and 1; n = 18) in high-grade serous carcinoma. (H: high p16 expression; L: low p16 expression). C, Patients with aberrant p53 expression (scores 0 and 3; n = 62) versus insignificant p53 expression (scores 1 and 2; n = 12) in high-grade serous carcinoma. D, Patients with high DEF6 expression (scores 2 and 3; n = 20) versus low DEF6 expression (scores 0 and 1; n = 16) in mucinous carcinoma. (H: high DEF6 expression; L: low DEF6 expression). E, Patients with high p16 expression (scores 2 and 3; n = 32) versus low p16 expression (scores 0 and 1; n = 3) in mucinous carcinoma. (H: high p16 expression; L: low p16 expression). F, Patients with aberrant p53 expression (scores 0 and 3; n = 17) versus insignificant p53 expression (scores 1 and 2; n = 19) in mucinous carcinoma. G, Patients with high DEF6 expression (scores 2 and 3; n = 6) versus low DEF6 expression (scores 0 and 1; n = 22) in endometrioid carcinoma. (H: high DEF6 expression; L: low DEF6 expression). H, Patients with high p16 expression (scores 2 and 3; n = 17) versus low p16 expression (scores 0 and 1; n = 11) in endometrioid carcinoma. (H: high p16 expression; L: low p16 expression). I, Patients with aberrant p53 expression (scores 0 and 3; n = 9) versus insignificant p53 expression (scores 1 and 2; n = 19) in endometrioid carcinoma. J, Patients with high p16 expression (scores 2 and 3; n = 30) versus low p16 expression (scores 0 and 1; n = 11) in clear cell carcinoma. (H: high p16 expression; L: low p16 expression). K, Patients with aberrant p53 expression (scores 0 and 3; n [file 13000_2016_518_MOESM1_ESM.zip › Figure_Supp1K.tif]
